# Effects of air pollution and seasons on health-related quality of life of Mongolian adults living in Ulaanbaatar: cross-sectional studies

**DOI:** 10.1186/s12889-017-4507-1

**Published:** 2017-06-23

**Authors:** Motoyuki Nakao, Keiko Yamauchi, Yoko Ishihara, Hisamitsu Omori, Dashtseren Ichinnorov, Bandi Solongo

**Affiliations:** 1 Department of Public Health, School of Medicine, Kurume University, 67 Asahimachi, Kurume, Fukuoka, 830-0011 Japan; 2 Department of Biomedical Laboratory Sciences, Faculty of Life Sciences, Kumamoto University, Kumamoto, Japan; 3 Department of Respiratory Medicine, Mongolian National University of Medical Sciences, Ulaanbaatar, Mongolia

**Keywords:** Health-related quality of life, Air pollution, Coal, Solid fuel, Respiratory symptoms, Mongolia

## Abstract

**Background:**

Ulaanbaatar, Mongolia, is known as severely air-polluted city in the world due to increased coal consumption in the cold season. The health effects of air pollution in Mongolia such as mortality, morbidity and symptoms have been previously reported. However, the concept of health-related quality of life (HR-QoL), which refers to the individual’s perception of well-being, should also be included as an adverse health outcome of air pollution.

**Methods:**

Surveys on the Mongolian people living in Ulaanbaatar were performed in the warm and cold seasons. Self-completed questionnaires on the subjects’ HR-QoL, data from health checkups and pulmonary function tests by respiratory specialists were collected for Mongolian adults aged 40–79 years (*n* = 666). Ambient PM2.5 and PM10 were concurrently sampled and the components were analyzed to estimate the source of air pollution.

**Results:**

In logistic regression analyses, respiratory symptoms and smoke-rich fuels were associated with reduced HR-QoL (> 50th percentile vs. ≤ 50th percentile). PM 2.5 levels were much higher in the cold season (median 86.4 μg/m^3^ (IQR: 58.7–121.0)) than in the warm season (12.2 μg/m^3^ (8.9–21.2). The receptor model revealed that the high PM2.5 concentration in the cold season could be attributed to solid fuel combustion. The difference in HR-QoL between subjects with and without ventilatory impairment was assessed after the stratification of the subjects by season and household fuel type. There were no significant differences in HR-QoL between subjects with and without ventilatory impairment regardless of household fuel type in the warm season. In contrast, subjects with ventilatory impairment who used smoke-rich fuel in the cold season had a significantly lower HR-QoL.

**Conclusions:**

Our study showed that air pollution in Ulaanbaatar worsened in the cold season and was estimated to be contributed by solid fuel combustion. Various aspects of HR-QoL in subjects with ventilatory impairment using smoke-rich fuels deteriorated only in the cold season while those with normal lung function did not. These results suggest that countermeasures or interventions by the policymakers to reduce coal usage would improve HR-QoL of the residents of Ulaanbaatar, especially for those with ventilatory impairment in the winter months.

**Electronic supplementary material:**

The online version of this article (doi:10.1186/s12889-017-4507-1) contains supplementary material, which is available to authorized users.

## Background

Global awareness of ambient air pollution has been growing due to its great risk to public health. World Health Organization (WHO) reported that annual concentration of ambient particulate matter air pollution was increased globally by 8% during the recent five years [1]. In particular, cities in low and middle income countries in the Western Pacific region suffered air pollution. Ulaanbaatar, the capital of Mongolia, is reported to be one of severely air-polluted cities in the world [2].

Health effects of particulate matter air pollution have been reported in terms of mortality and morbidity of cardiopulmonary diseases, prevalence of respiratory symptoms and hospital admission [3–5]. In Mongolia, air pollution has been reported to enhance urinary excretion of 1-hydroxypyrene in children, which is a biomarker for polyaromatic hydrocarbons (PAHs) [6], byproducts derived from coal combustion exposure [7]. Dashdendev et al. reported that carbon monoxide pollution contributed to the deterioration of lung function in Mongolian children [8]. Although the health effects of air pollution including mortality, morbidity, symptoms and physiological functions have been previously reported in Mongolia [9], the concept of health-related quality of life (HR-QoL), which refers to the individual’s perception of well-being, should also be considered as an adverse health outcome [10]. Our previous study reported that the short-form 36 health survey (SF-36v2) Mongolian version and COOP/WONCA charts are valid and reliable questionnaires to evaluate HR-QoL of Mongolian adults [11]

In Mongolia, in addition to its geographical feature as a basin that tends to trap polluted air, the increased consumption of solid fuels has contributed significantly to air pollution in Ulaanbaatar [12]. Mass migration from the rural areas into Ulaanbaatar has risen over the last two decades, resulting in an increase in population and population density [13]. Migrated people built ger, the traditional nomadic dwelling of Mongolians, in the perimeter of the urban area. The ger district is heavily dependent on solid fuels such as coal and wood as household fuel for heating and cooking. This causes air pollution that spans a considerably long duration in Mongolia, especially in the cold season. Air pollution in Ulaanbaatar has also been attributed to the heat-only boilers and coal fired thermal power plants [14]. These sources of air pollutants have frequently caused Ulaanbaatar to be covered in smog in the cold season.

In the current study, health surveys targeted at Mongolian subjects over 40 years of age were carried out in the urban area and ger district of Ulaanbaatar during the cold and warm seasons. At the same time, ambient particulate matter and aerodynamic particle size distribution were measured, and the component of PM2.5 was analyzed to identifying the source of pollution (source appointment) in Ulaanbaatar to assess the impact of air pollution on HR-QoL in Mongolian adults.

## Methods

### Study design

Institutional based cross-sectional study was conducted among Mongolian adults aged between 40 and 79 years at eight facilities (two hospitals and six community clinics) in Ulaanbaatar. The questionnaires were administered to study participants in September of 2012 and July of 2013 (warm season) and March and December of 2013 (cold season); clinical procedures were also conducted at these times. Air sampling took place in September 2012, March 2013, July 2013, and December 2013*.*. Health survey was conducted at least two hospitals or community clinics in both ger district and the urban area for each study.

### Subjects

**Fig. 1:**
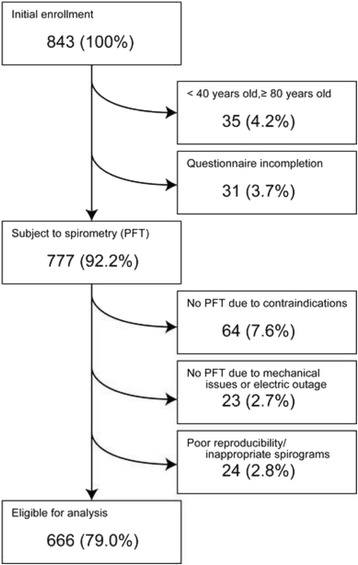
Flow chart describing the process for screening the sample available for further analysis

Participants were recruited by announcements advertising the health survey. Subjects received a self-administered questionnaire containing questions pertaining to age, gender, occupation, respiratory symptoms, and medical history. At the time of questionnaire administration, height and body weight were measured, and medical interviews, auscultation, and pulmonary function tests were conducted by respiratory specialists. The participants only filled out questionnaires and underwent exams one time. The study initially included 843 male and female community volunteers. Of these, 35 (4.2%) subjects were excluded from the analysis due to their age lying beyond the targeted range. 31 (3.7%) subjects were excluded due to incomplete questionnaires. The remaining 777 (92.2%) were subject to pulmonary function tests. However, the physician diagnosed 64 (7.6%) subjects to be contraindicated for spirometry due to conditions such as severe respiratory infections, active tuberculosis, post-pneumonectomy, or high blood pressure. Furthermore, spirometry on 23 (2.7%) subjects could not be performed due to mechanical issues or electric outage. Finally, 24 (2.8%) subjects were excluded from analysis due to other reasons such as poor reproducibility and/or inappropriate spirograms. Consequently, 666 (79.0%) of the remaining subjects were eligible for further analysis (Fig. 1).

### Health survey

Medical interviews and auscultation were conducted by physicians. A pre-bronchodilator pulmonary function test using the HI-105 spirometer (CHEST M.I., Inc., Tokyo, Japan) was conducted by respiratory specialists. Spirometers were calibrated before each survey. At least three spirograms were obtained, and the highest vital capacity (VC), forced expiratory volume in one second (FEV_1_), and forced ventilatory capacity (FVC) values were applied for diagnosis. Respiratory specialists assessed the reproducibility and quality of spirograms (volume-time curve and flow-volume loop). The invalid spirograms showing artefacts, insufficient inspiration or blow-out were excluded from analysis. Predicted values of FEV_1_ and FVC were determined using the GLI2012 reference equations for North East Asians [15]. Subjects with FEV_1_/FVC ratio < 70% and/or VC < 80% of its predicted value were classified as “ventilatory impairment”. The questionnaire is a self-completed booklet containing questions on age, gender, occupation, respiratory symptoms, as well as the SF-36v2 and COOP/WONCA charts. Responses to the SF-36v2 were on three-, five- or six-point ordinal scales, from which we calculated the eight subscales (physical functioning; role limitations due to physical health problems (role-physical); bodily pain; general health perceptions; vitality; social functioning; role limitations due to emotional problems (role-emotional); and mental health) from 0 to 100 points (100 = best) according to the scoring manual [16–18]. Responses to the COOP/WONCA charts comprised of eight items (physical fitness; feelings; daily activities; social activities; change of health; overall health; pain; quality of life) that were scored on a five-point ordinal scale ranging from 1 to 5 (1 = best, 5 = worst) [19].

### Sampling and analysis of the constituents of ambient particulate matters

Ambient air was sampled in the center of Ulaanbaatar city at 47°54′53.0″N 106°55′24.2″E, away from any smoke generating facilities during 14 Sep – 19 Sep, 2012 (warm season); 1 Mar – 6 Mar, 2013 (cold season); 27 Jun – 3 Jul, 2013 (warm season); 27 Nov – 6 Dec, 2013 (cold season). Fine particles with a diameter of 2.5 μm or less (PM2.5) were collected on a polytetrafluoroethylene (PTFE) filter using FRM-2000 (Thermo Fisher Scientific Inc., Waltham, MA, USA) at a flow rate of 16.7 L/min. Coarse particles with a diameter of 10 μm or less (PM10) were collected on a glass-fiber filter using HV-500F (SIBATA SCIENTIFIC TECHNOLOGY LTD., Tokyo, Japan) at a flow rate of 500 L/min. Sampled filters were stored in hermetically sealed plastic bags at −20 °C. Used filters were weighed, followed by conditioning at 21.5 ± 1.5 °C and 35.0 ± 5.0% relative humidity using an electric analytical scale (ME-5F, Sartorius AG, Göttingen, Germany). Mass concentrations were calculated by subtracting the pre-sampling filter weight from the post-sampling filter weight for the same filter. Daily average concentrations were calculated from 9 am to 9 am the next morning. Aerodynamic particle size distribution was measured continuously using an Aerodynamic Particle Sizer (APS) spectrometer 3321 (TSI Inc., Shoreview, MN, USA). Data including the number, surface area, and mass of the particles of aerodynamic diameter from 0.523 to 20 μm were recorded during the health survey in July and December. Chemical species of PM2.5 we analyzed were as follows: carbonaceous fractions including organic carbon (OC), elemental carbon (EC); ions including chloride (Cl^−^), nitrate (NO_3_^−^), sulfate (SO_4_^2−^), sodium (Na^+^), ammonium (NH_4_^+^), potassium (K^+^), magnesium (Mg^2+^), calcium (Ca^2+^); elements including sodium (Na), aluminum (Al), silicon (Si), potassium (K), calcium (Ca), scandium (Sc), titanium (Ti), vanadium (V), chromium (Cr), manganese (Mn), iron (Fe), cobalt (Co), nickel (Ni), copper (Cu), zinc (Zn), arsenic (As), selenium (Se), rubidium (Rb), molybdenum (Mo), antimony (Sb), cesium (Cs), barium (Ba), lanthanum (La), cerium (Ce), samarium (Sm), hafnium (Hf), tantalum (Ta), tungsten (W), lead (Pb), thorium (Th). These analyses were carried out by Fujitsu Quality Laboratory, Inc. (Kanagawa, Japan). In brief, carbonaceous fractions, ions and elements were analyzed by the thermal optical reflectance method using Sunset Laboratory Inc. (Tigard, OR, USA) model CAA-202 M-D, ion chromatography using Metrohm AG (Herisau, Switzerland) model IC850 and Inductively Coupled Plasma-Mass Spectrometry (ICP-MS) using Agilent Technologies, Inc. (Santa Clara, CA, USA) model 7700×. All procedures were performed according to the manufacturer’s and government instructions for component analysis of ambient fine particulate matter [20].

### Source appointment of PM2.5 particles

Source appointment of PM2.5 particles was carried out based on the EPA Positive Matrix Factorization (PMF) 5.0 Fundamentals and User Guide [21]. We measured two carbonaceous fractions, eight ions and 30 elements. Of these, Mg^2+^, Cr, Mo, Ta, Cd were excluded from analysis due to missing values and data being below the limit of detection. Three ions (Na^+^, K^+^, and Ca^2+^) were also excluded to avoid double counting. Additionally, Co was excluded due to low signal-to-noise ratio (S/*N* = 0.1), and Ba was also excluded since the correlation between the observed and predicted value was extremely low (r^2^ < 0.05). PM2.5 mass concentration was also included in the analysis and was categorized as “weak” species because it was sum total of variables including carbonaceous fractions, ions and elements. Missing values were substituted with the median of the same species of the same season (warm or cold). The original values measured were used in the data analysis despite being below the detection limit. Consequently, PM2.5 mass, carbonaceous fractions, four ions and 25 elements were included in the receptor model developed by EPA PMF v5.0. The PMF was carried out using the robust mode. Input data consisted of two files; concentrations of chemical species and equation-based uncertainties. Uncertainty was calculated by taking into consideration of concentration, method detection limit and error fractions (5–25%). Error fractions were determined by concentration, geometric mean values and geometric standard deviation of samples as follows;

$$ \mathrm{Cr}= Concentration/ Geometric\  mean $$

If Cr ≥ σ^2^, error fraction is 0.05; σ^2^ > Cr ≥ σ, error fraction is 0.10; σ > Cr ≥ σ^−1^, error fraction is 0.15; σ^−1^ > Cr ≥ σ^−2^, error fraction is 0.20; Cr < σ^−2^, error fraction is 0.25, where σ = geometric standard deviation.

Uncertainties were calculated as follows:

$$ \mathrm{Uncertainty}=\sqrt{{\left( Error\  fraction\times Concentration\right)}^2+{\left(0.5\times method\  detection\  limit\right)}^2} $$

Uncertainties for data below the detection limit were determined by 5/6 of the method detection limit. For missing data, uncertainty was calculated by four times the median value of measured data in the same season. PMF is a multivariate receptor model resolved by minimizing the sum of the squared and scaled residuals (Q). The behavior of the Q value as a function of the rotational parameter Fpeak has been used to provide insight into the rotational stability of modeling results, with a lower Q value corresponding to a more stable PMF solution [22]. In this study, bootstrap runs at Fpeak value of −0.5 proved to map better than those of the base run bootstrap model. Samples with large scaled residuals greater than 3 were multiplied by factors of 2 to 8 to decrease their weight in the model fit [23].

### Independent variables

For logistic regression analysis, age (years old), gender (0, female; 1, male), body mass index (BMI)(0, BMI ≥ 25; 1, BMI < 25 kg/m^2^), ventilatory impairment (0, FEV_1_/FVC ratio ≥ 0.7; 1, FEV_1_/FVC < 0.7), current smoking status (0, never or ex-smoker; 1, current smoker), the PM2.5 mass concentration (μg/m^3^), time spent outdoors (hours), household fuel type (0, smoke-free fuel such as gas and electricity; 1, smoke-rich fuel such as coal and wood), self-reported asthma (0, none; 1, asthma), respiratory symptoms (Q1, Does the weather affect your cough?; Q2, Have you ever coughed up sputum from your chest when you do not have a cold?; Q3, Do you usually cough up sputum from your chest first thing in the morning?; Q4, How frequently do you wheeze?; Q5 Do you have or have you had any allergies?; Q6: Do you suffer from any infectious diseases? (0, No (Q1–3, 5 and 6) or Never (Q4); 1, Yes (Q1–3, 5 and 6) or Occasionally or more often (Q4)) were applied as independent variables. All independent variables were included in each model. We choose these variables a priori because older age is associated with lung function; smoking habit has a close relation to gender; and PM2.5 level, time spent outdoors and household fuel type are related to the exposure level to air pollution [24]. BMI is reported to be associated with the severity of obstructive lung disease, which affects patients’ HR-QoL [25]. Self-reported asthma and respiratory symptoms were considered to have relationships with decreased pulmonary function.

### Data handling and statistical analyses

All data were anonymized and managed as electronic data for the analysis. We dichotomized the COOP/WONCA chart scores and SF-36v2 subscale scores as dependent variables (0 for ≤50 percentiles of the scores (less affected); 1 for >50 percentiles (more affected)) to represent more interpretable for logistic regression analyses. The odds ratios (OR) were adjusted by independent variables (age, gender, BMI, ventilatory impairment, current smoking status, PM2.5 mass concentration, household fuel type, self-reported asthma, time spent outdoors and six kinds of respiratory symptoms) (see *Independent variables*). For age and PM2.5 mass concentration, the ORs were presented as incremental difference of 10 years of age and of 10 μg/m^3^ of PM2.5. The omnibus tests of model coefficient and goodness-of-fit test analyzed by Hosmer-Lemeshow statistics were carried out to assess validity of the logistic model. Participants were stratified by the household fuel type and seasons for the comparison of HR-QoL scores between the subjects with and without ventilatory impairment because the primary target of air pollution was respiratory tract and the household fuel type and seasons were associated with air pollution. Statistical analyses including Welch’s *t*-test for parametric analysis of two groups and logistic regression analysis were performed using the statistical software package JMP version 12 (SAS Institute Inc., Cary, NC, USA) and SPSS ver. 21 (IBM Corporation, Armonk, NY, USA). *P* values of less than 0.05 were considered statistically significant.

## Results

### Characteristics of participants

Table 1 shows the characteristics of participants. The ratio of male to female participants was approximately 1:1.75, and the mean age with standard deviation (SD) was 54.0 ± 10.0 years old. Mean BMI with SD was 27.6 ± 5.0, and current smoking rate was 26.1%. Mean time spent outdoors with SD was 6.0 ± 4.9 h. The prevalence of ventilatory impairment and self-reported asthma were 24.6% and 22.3%, respectively.

**Table 1:** Characteristics of participants

*N* = 666				
Characteristics			Smoke-rich fuel user	
Gender	N	%	N	%
Overall	666	100	403	61.9
Male	242	36.3	155	65.4
Female	424	63.7	248	59.9
Age (years)				
Overall (mean ± SD)	54.0 ± 10.0		-	
Smoke-rich fuel user	53.3 ± 9.5		-	
Smoke-free fuel user	55.3 ± 10.7		-	
BMI (kg/m^2^)				
Overall (mean ± SD)	27.6 ± 5.1		-	
Smoke-rich fuel user	27.7 ± 5.2		-	
Smoke-free fuel user	27.7 ± 4.9		-	
Smoking status	N	%	N	%
Never-smoker	447	67.1	257	59.4
Ex-smoker	45	6.8	28	63.4
Current smoker	174	26.1	118	67.8
Time spent outdoors (hours)				
Overall (mean ± SD)	6.0 ± 4.9		-	
Smoke-rich fuel user	6.0 ± 5.0		-	
Smoke-free fuel user	6.2 ± 4.7		-	
Prevalence	N	%	N	%
Ventilatory impairment	164	24.6	97	61.0
Self-reported asthma	146	22.3	97	68.3

### Multivariate analysis to assess the factors associated with HR-QoL of participants

Logistic regression analyses were carried out using age, gender, BMI, pulmonary function, smoking status, PM2.5 mass concentration, household fuel type, self-reported asthma, time spent outdoors and six kinds of respiratory symptoms as predictive variables, with the COOP/WONCA chart items and subscales of SF-36v2 as dependent variables (Table 2, Additional file 1 Table S1). The omnibus tests of model coefficient showed significant (*P* < 0.001) for all models. Goodness-of-fit were analyzed by Hosmer-Lemeshow statistics, and the *P* values were well above 0.05 for all models. The physical fitness item of the COOP/WONCA chart and the physical functioning subscale of the SF-36v2 were getting worse in association with 10-year increment of age while the feelings, daily activities and quality of life items of the COOP/WONCA chart and the mental health subscale of the SF-36v2 were getting better with age. The physical fitness, feelings, overall health and pain items of the COOP/WONCA chart and the bodily pain, general health perceptions, vitality, social functioning and mental health subscales of the SF-36v2 were better in male subjects. The social activities item of the COOP/WONCA chart and the social functioning and mental health subscales of the SF-36v2 were worse in the subjects with BMI less than 25 while the change of health item of the COOP/WONCA chart was better in those with BMI less than 25. Increment of 10 μg/m^3^ of PM2.5 mass concentration was only associated with the worse physical fitness item of the COOP/WONCA chart. Subjects using smoke-rich fuels showed significant decline in the overall health, pain and quality of life items of the COOP/WONCA chart and the role-physical, bodily pain, general health perceptions, vitality, role-emotional and mental health subscales of SF-36v2. Self-reported asthma seemed to affect the feelings and overall health items of the COOP/WONCA chart and the social functioning subscale of the SF-36v2. The daily activities item of the COOP/WONCA chart and the physical functioning, general health perceptions and vitality subscales of the SF-36v2 were better in association with time spent outdoors. With respect to respiratory symptoms, the daily and social activities and pain items of the COOP/WONCA chart and the physical functioning subscale of the SF-36v2 worsened in association with presenting the symptom Q1 (Weather affects cough). The feelings, daily activities and overall health items of the COOP/WONCA chart and the physical and social functioning, bodily pain, general health perceptions and role-emotional subscales of the SF-36v2 declined in subjects presenting with the symptom Q2 (Coughing up phlegm without having a cold). The symptom Q4 (Frequent wheeze) were only associated with the deteriorated general health perception subscale of the SF-36v2. The symptom Q6 (Infectious disease) was concerned with worsened scores of the feelings, daily activities, change of health, pain and quality of life items of the COOP/WONCA chart and the bodily pain, general health perceptions and vitality subscales of the SF-36v2. The symptoms Q3 (Coughing up phlegm first thing in the morning) and Q5 (Allergy) were not associated with any scores of the COOP/WONCA and SF-36v2.

**Table 2:** Association between the factors regarding indoor and outdoor air quality with HR-QoL of Mongolian subjects (*n* = 666) in Ulaanbaatar in 2012–2013

Questionnaire	Dependent variables	Median (cutoff)	Independent variables			
			Higher PM2.5 (10 μg/m^3^)		Smoke-rich household fuel	
			Crude OR (95% C.I.)	Adjusted OR (95% C.I.)	Crude OR (95% C.I.)	Adjusted OR (95% C.I.)
COOP/WONCA	Physical fitness	1.0	1.06 (1.01–1.12)	1.12 (1.05–1.18)	1.01 (0.73–1.38)	1.13 (0.80–1.61)
	Feelings	2.0	1.05 (1.00–1.10)	1.02 (0.96–1.09)	1.20 (0.86–1.67)	1.36 (0.93–1.99)
	Daily activities	2.0	1.01 (0.95–1.06)	0.97 (0.90–1.03)	1.29 (0.90–1.84)	1.33 (0.89–1.99)
	Social activities	1.0	1.03 (0.97–1.08)	0.99 (0.93–1.05)	1.28 (0.92–1.77)	1.33 (0.93–1.91)
	Change of health	3.0	0.96 (0.90–1.03)	0.92 (0.85–1.00)	1.47 (0.94–2.30)	1.52 (0.92–2.49)
	Overall health	4.0	1.01 (0.96–1.06)	1.00 (0.94–1.06)	1.28 (0.94–1.76)	1.46 (1.02–2.07)
	Pain	3.0	1.00 (0.94–1.05)	0.98 (0.92–1.05)	1.38 (0.98–1.95)	1.58 (1.07–2.33)
	Quality of life	2.0	1.06 (1.01–1.12)	1.02 (0.96–1.08)	1.58 (1.14–2.19)	1.68 (1.17–2.41)
SF-36	Physical functioning	65.0	0.99 (0.94–1.04)	0.98 (0.92–1.04)	1.29 (0.94–1.78)	1.39 (0.97–2.00)
	Role physical	62.5	0.98 (0.93–1.03)	0.95 (0.90–1.01)	1.68 (1.22–2.32)	1.63 (1.15–2.31)
	Bodily pain	61.0	0.96 (0.91–1.01)	0.92 (0.87–0.98)	1.68 (1.22–2.31)	1.97 (1.37–2.84)
	General health perceptions	50.0	1.00 (0.95–1.05)	0.98 (0.92–1.04)	1.48 (1.08–2.03)	1.78 (1.24–2.55)
	Vitality	62.5	1.00 (0.95–1.05)	0.98 (0.92–1.04)	1.55 (1.12–2.13)	1.52 (1.07–2.16)
	Social functioning	75.0	0.99 (0.94–1.04)	0.96 (0.90–1.02)	1.32 (0.95–1.83)	1.30 (0.90–1.86)
	Role emotional	66.7	1.00 (0.95–1.05)	0.97 (0.92–1.03)	1.82 (1.31–2.53)	1.93 (1.35–2.76)
	Mental health	70.0	1.04 (0.98–1.09)	1.00 (0.94–1.06)	1.76 (1.28–2.42)	1.69 (1.19–2.41)

The COOP/WONCA items were scored on a five-point ordinal scale ranging from 1 to 5 (lower is better), and responses to the SF-36 were calculated the eight subscales from 0 to 100 points (higher is better). The median value of the scores were used for cutoff point. The COOP/WONCA chart and SF-36 subscale scores were dichotomized as dependent variables (0 for ≤50 percentiles of the scores (less affected); 1 for >50 percentiles (more affected)). Odds ratios were adjusted by age, gender, BMI, ventilatory impairment, smoking status, self-reported asthma, time spent outdoors, and six kinds of respiratory symptoms in addition to PM2.5 concentration and household fuel type.

### Ambient air pollution and the source appointment of PM2.5 in Ulaanbaatar

**Fig. 2:**
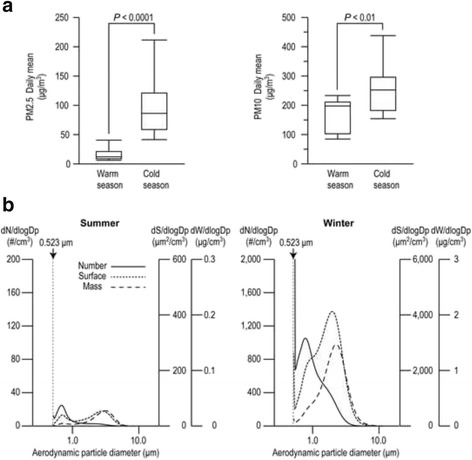
Seasonal differences of particulate air pollution level in Ulaanbaatar, Mongolia. Ambient air was sampled during the health survey. Daily mean of mass concentrations of PM2.5 (left panel of **a**) and PM10 (right panel of **b**) were presented as boxplots. Each boxplot shows the most extreme values of the daily mean PMs (maximum and minimum values), the lower and upper quartiles, and the median. The bottom, middle and top lines of each box correspond to the 25th percentile, median, and the 75th percentile, respectively. Seasonal variations of particle size distributions during the health survey (summer and winter) in Mongolia were presented (**b**); solid line, number concentration; dotted line, surface area; dashed line, mass concentration. Particle size is expressed in logarithmic scale

Ambient air pollution was measured as PM2.5 and PM10. Daily means of PM2.5 and PM10 during the health survey in the cold season [94.3 ± 45.4 μg/m^3^ (mean ± SD, PM2.5), 248.6 ± 79.5 μg/m^3^ (PM10)] were significantly higher than that of the warm season [15.3 ± 9.5 μg/m^3^ (PM2.5) 167.8 ± 54.6 μg/m^3^ (PM10)] (Fig. 2a). Particle size distribution measured by APS is shown in Fig. 2b. Values representing the number of particles, surface area, and mass in winter were much higher than that in summer. The peak in particle number, surface area, and mass distribution in the summer occurred at approximately 0.7 μm, 3.0 μm, and 3.5 μm in diameter respectively. In the winter, the peak in particle number, surface area, and mass distribution occurred at approximately 0.7 μm, 2.0 μm, and 2.3 μm in diameter respectively. In addition to these peaks, the peaks at 0.8 μm in diameter for the surface area and mass also occurred in both seasons. The highest point of the number and surface area in winter was 0.523 μm in diameter, which is the lowest detection limit.

**Fig. 3:**
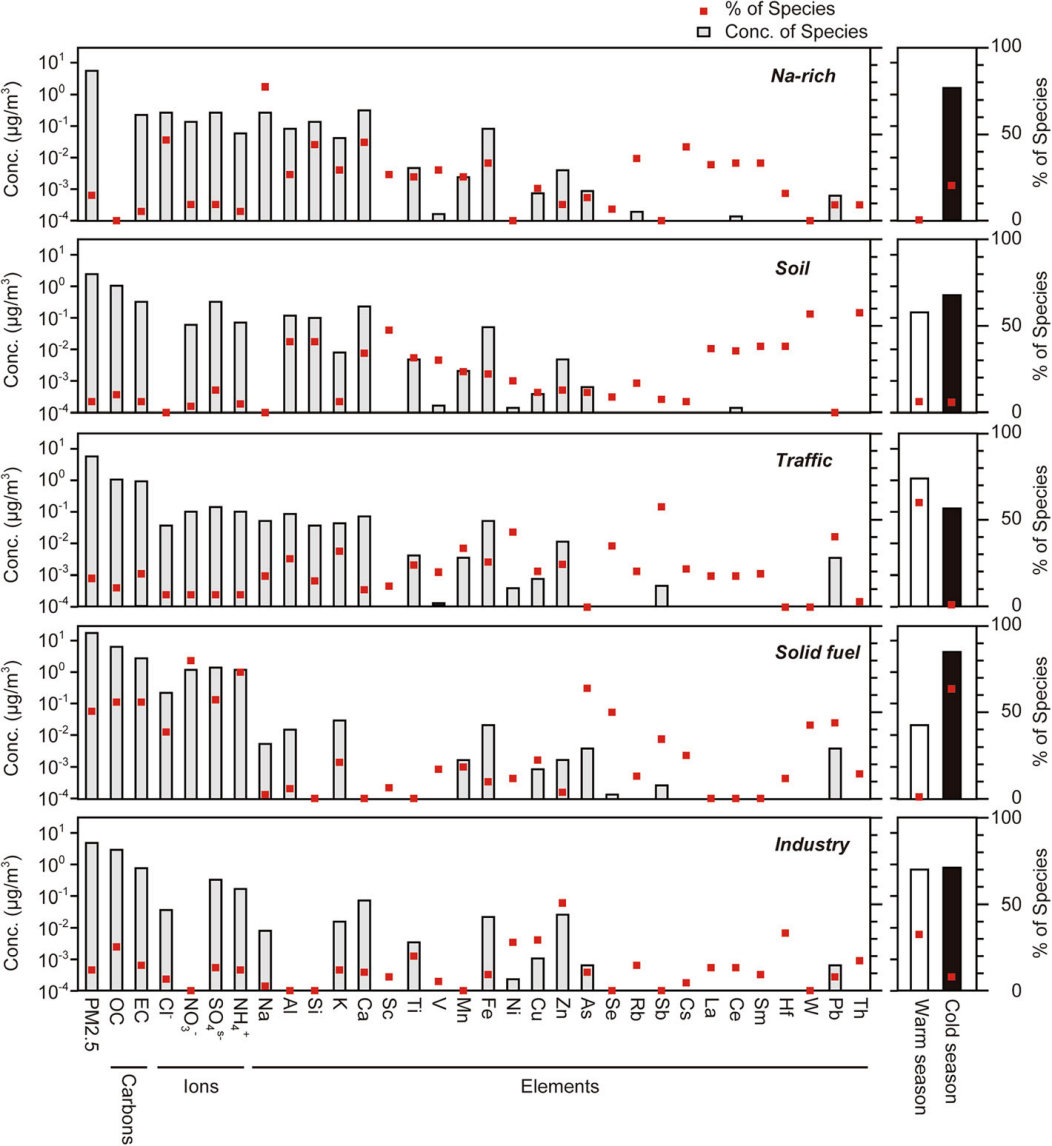
Source profiles and contributions determined by PMF solutions for PM2.5 in Ulaanbaatar, Mongolia. Left panel shows the source profiles of each species. Shaded bars represent the concentration of each species and red squares represent the percentage of each species sum. Right panel shows the factor contribution to PM2.5 in each season. Blank bars and solid bars represent the concentrations of each factor in the warm season and cold season, respectively. Red squares represent the percentage of the contribution of each factor in each season

Profiles identified for the five-factor solution included: (1) Na-rich (Na and Cl^−^); (2) Soil; (Al and Si) (3) Traffic (Sb and Mn); (4) Solid fuel (Carbonaceous fraction, ions, As and Pb); (5) Industry (Zn, Ni and Ti) (Fig. 3). PM2.5 mass, carbonaceous fractions, ions except for sulfate, Na, Al, Si, Ca, Sc, Ti, V, Mn, Fe, Cu, As, Se, Rb, Cs, La, Ce, Sm, Hf and Th, and were well predicted (i.e., r^2^ (observed/predicted) ≥ 0.8). Sulfate ions, Zn, Sb and W were moderately predicted (i.e., 0.8 > r^2^ ≥ 0.6). K, Ni and Pb were poorly predicted (i.e., r^2^ < 0.6). When the factor number was changed from five to six, Q/Q_expected_ decreased from 1.89 to 1.75, which was a larger decrease than when the factor number was changed from four to five (1.75 to 1.64). Since a smaller decrease in Q indicates that the possibility of too many factors being fit, five factors may be the optimal solution. Na-rich, Soil, Traffic, Solid fuel and Industry factors were mapped 88%, 99%, 89%, 99% and 89% of bootstrap run, respectively. The bootstrap results improved after a factor rotation of the Fpeak value at −0.5. Mapping of Fpeak boot factors to base factors were as follows; Na-rich, Soil, Traffic, Solid fuel and Industry factors were mapped 95%, 98%, 96%, 100% and 97% of bootstrap run, respectively. Fig. 3 indicates that the contributions by Traffic and Industry factors were high in the warm season, and that Soil factor contributed similarly in both the warm and cold season. These factors contributed relatively little to the PM2.5 mass. Na-rich and Solid fuel factors contributed predominantly in the cold season. Over half of the PM2.5 mass was attributed to Solid fuel factor.

### Association of household fuel type and season with HR-QoL of Mongolian subjects with ventilatory impairment

To examine the association of household fuel type and season with HR-QoL of Mongolian subjects, we compared HR-QoL measured by the SF-36v2 and COOP/WONCA charts between subjects with normal lung functions and those with ventilatory impairment, when the subjects were stratified by season and household fuel type. The daily activities and pain item measured by the COOP/WONCA charts were significantly worse only in the subjects with ventilatory impairment using smoke-rich fuels in the cold season. The physical functioning role physical, bodily pain, general health perceptions, and social functioning subscales of the SF-36v2 were also significantly worse in subjects with ventilatory impairment using smoke-rich fuels in the cold season. Only the role-emotional subscale was significantly worse in the subjects with ventilatory impairment using smoke-free fuels in the cold season. No significant differences between the subjects with normal lung function and those with ventilatory impairment were found in the warm season regardless of household fuel type.

## Discussion

We conducted the health survey on 666 Mongolian subjects living in Ulaanbaatar (Fig. 1). We observed an inverse association between respiratory symptoms and smoke-rich fuels and HR-QoL. PM2.5 and PM10 were much higher in the cold season than in the warm season. The PMF receptor model revealed that the high concentration of PM2.5 in the cold season was estimated to be attributable to solid fuel combustion. We thus investigated the difference in HR-QoL between the subjects with and without ventilatory impairment after the stratification by season and household fuel type. There was no significant difference in HR-QoL between subjects with and without ventilatory impairment regardless of household fuel type in the warm season. In contrast, subjects with ventilatory impairment using smoke-rich fuel in the cold season reported worse scores of the various aspects of HR-QoL when compared to the subjects without ventilatory impairment.

Ulaanbaatar, Mongolia is one of severely air-polluted cities in the world [2]. One reason is the increased consumption of coal during winter due to the population influx to Ulaanbaatar and subsequent expansion of the ger district. Another reason is attributed to its geographical feature as a basin [12]. Recently, Jadambaa et al. [9] reported that air pollution, metals, tobacco smoke and toxic chemicals are environmental risk factors for the Mongolian population involved in the development of cardiopulmonary diseases in adults and neurodevelopmental and respiratory disorders in children. In addition to cardiopulmonary effects, a decrease in HR-QoL is reported to be an adverse health effect as a result of exposure to air pollution [26]. In the current study, we performed logistic regression analyses to determine the factors associated with HR-QoL in Mongolian subjects (Table 2). Higher PM2.5 concentration was only associated with a worse physical fitness item of the COOP/WONCA chart. The use of smoke-rich household fuel seemed to affect the overall health, pain and quality of life items of the COOP/WONCA chart and all subscales except for physical and social functioning of the SF-36v2. These results suggest that the type of household fuels was more strongly associated with HR-QoL than was PM2.5 concentration.

We monitored the ambient PM2.5, PM10 levels and particle size distribution during the health survey. In the cold season, PM2.5 and PM10 were much higher than in the warm season (Fig. 2a). These results were consistent with a previous report [27]. When compared with the current WHO guidelines on the 24-h average of PM2.5 (25 μg/m^3^) and PM10 (50 μg/m^3^), our results showed that these readings were three to five times higher in the cold season. These results seemed to be due to the increased consumption of smoke-rich fuel in the ger district during the winter months because of poor infrastructure catering to electricity and gas in the ger district in Ulaanbaatar [28]. Size distribution of particles showed a distinct peak at less than 5 μm in diameter in the winter (Fig. 2b). Particles less than 7 μm in diameter can be inhaled into the lungs, and fine particles can reach the alveolar, which is sensitive to foreign particles. Moreover, fine particles constitute a larger surface area than the same mass of coarse particles. Consequently, fine particles can transport more toxic compounds attached on its surface to deeper parts of the lungs than coarse particles per unit weight [29]. Source appointment of PM2.5 particles was estimated using the PMF receptor model (Fig. 3). The model solution contains five factors: (1) Na-rich; (2) Soil; (3) Traffic; (4) Solid fuel; (5) Industry. The origin of the Na-rich factor was unclear because Mongolia is an inland state (there is no sea salt). In addition to Na and Cl^−^, this factor also contained high contribution of Al and Si as well as Soil factor. Na-rich factor, unlike soil factor, showed high contribution in the cold season. Only the samples obtained on a snowy day in December showed extremely high contribution by the Na-rich factor. We speculate that this factor might be derived from drifting snow or snow-melting agents (e.g., sodium chloride) with soil/dust from the land surface. The contribution of Soil factor was relatively small compared to other anthropogenic sources, although there are many unpaved roads and exposed land surfaces in Ulaanbaatar [14]. Major constituents of soil contribute largely to coarse particles rather than fine particles [30, 31]. This could be the reason why the high concentration of PM10 was observed even in the warm season where the coal consumption and PM2.5 concentration were markedly decreased. The number of cars in Mongolia drastically increased five-fold from 2000 to 2011, leading to chronic traffic congestion in the urban area of Ulaanbaatar [32]. Traffic factor was characterized by Sb, a component of brake lining [33]. This factor also contained nitrate ions, which is reported to be associated with traffic emissions [34]. A large part of the air pollution in Ulaanbaatar was reported to be caused by coal burning in the ger district and in coal-fired power plants [28, 35]. The characteristic of the source profile of Solid fuel factor was consistent with a previous study [36]. Guttikunda reported that approximately five tons of coal and 3 m^3^ of wood are used domestically in each ger annually, and nearly 60% of the coal is consumed between November and February [37]. Our results were consistent with a previous report indicating that the highest source contributor of PM2.5 in Ulaanbaatar was from a coal burning source as it was dominated by black carbon and significant Sulphur components, although the report did not show fractionated carbon, ions and toxic heavy metals [35]. The concentration of Industry factor was almost the same in both seasons, and was much lower than the Solid fuel factor and Traffic factor in the cold season.

Subjects using smoke-rich fuels and presented with respiratory symptoms showed a trend of poorer HR-QoL compared to those using smoke-free fuels (Table 2). A higher contribution of Solid fuel factor was also found in the cold season (Figs. 2 and 3). The effects of chronic respiratory diseases on the HR-QoL were previously reported [38, 39]. We therefore assessed the association of season and household fuel type with HR-QoL in subjects with ventilatory impairment. All dimensions of HR-QoL in subjects with ventilatory impairment were compared with those with normal lung function after the stratification of subjects by season and household fuel type (Table 3). Subjects with ventilatory impairment who used smoke-rich fuel showed a significant lower HR-QoL scores only in the cold season compared to those with normal lung function. Since PM2.5 concentration or usage of smoke-rich household fuel were strongly associated with ambient temperature (i.e. PM2.5 and usage of smoke-rich fuels were increased in the cold season), we could not rule out the possibility that colder temperature affected HR-QoL of the subjects with ventilatory impairment. Nevertheless, the subscales of HR-QoL were not significantly different between subjects with and without ventilatory impairment even in the cold season if the subjects used smoke-free fuel except for role-physical subscale of the SF-36v2 while subjects using smoke-rich fuel with ventilatory impairment showed significant lower HR-QoL in many subscales. These results suggest that the decline in HR-QoL could be attributable not to cold weather but to worsened air pollution. Domestic use of unprocessed solid fuels not only cause outdoor air pollution but also indoor air pollution [40]. There are many reports that indoor air pollution plays a role in the development and exacerbation of cardiopulmonary diseases [41–44]. Consequently, our findings suggest that indoor air pollution derived from domestic coal combustion is associated with deterioration of HR-QoL in subjects with ventilatory impairment in the cold season. Alexander et al. reported that a reduction of indoor air pollution improved respiratory health-related quality of life [45]. Although we did not monitor air pollution inside the ger in which the residents depend on solid fuels for domestic use, Enkhbat et al. previously reported that the PM2.5 mass concentration in a ger is more than two times higher than that in an apartment in Ulaanbaatar [46].

**Table 3:** Comparison of the HR-QoL between Mongolian subjects with and without ventilatory impairment when the subjects were stratified by season and household fuel type

Questionnaire	Season	Warm						Cold	
	Household fuel type	Smoke-free (*n* = 120)		Smoke-rich (*n* = 210)		Smoke-free (*n* = 128)		Smoke-rich (*n* = 193)	
	Ventilatory impairment	(−) *n* = 87	(+) *n* = 33	(−) *n* = 161	(+) *n* = 49	(−) *n* = 99	(+) *n* = 29	(−) *n* = 145	(+) *n* = 48
	Item/ subscale								
COOP/WONCA [Mean ± SD]	Physical fitness	1.8 ± 1.0	2.2 ± 1.4	1.8 ± 1.1	2.0 ± 1.3	1.9 ± 1.2	2.3 ± 1.2	2.2 ± 1.3	2.6 ± 1.6
	Feelings	2.2 ± 1.0	2.0 ± 1.1	2.4 ± 1.2	2.3 ± 1.2	2.2 ± 1.1	2.4 ± 1.1	2.3 ± 1.1	2.5 ± 1.1
	Daily activities	1.7 ± 0.8	1.7 ± 1.1	2.0 ± 1.0	2.0 ± 1.1	1.9 ± 0.9	1.9 ± 0.9	1.9 ± 0.9	2.3 ± 1.1*
	Social activities	1.5 ± 0.8	1.4 ± 0.7	1.7 ± 0.9	1.6 ± 0.9	1.7 ± 1.1	1.5 ± 0.8	1.5 ± 0.8	1.8 ± 1.0
	Change of health	2.6 ± 0.9	2.7 ± 0.8	2.7 ± 1.0	2.7 ± 0.9	2.5 ± 1.0	2.4 ± 1.0	2.7 ± 0.8	2.9 ± 0.9
	Overall health	3.2 ± 0.9	3.5 ± 0.9	3.4 ± 0.8	3.5 ± 0.6	3.3 ± 0.9	3.3 ± 1.0	3.4 ± 0.8	3.5 ± 0.9
	Pain	2.5 ± 1.1	2.6 ± 1.4	2.9 ± 1.2	2.7 ± 1.3	2.5 ± 1.3	2.8 ± 1.2	2.6 ± 1.2	3.1 ± 1.2*
	Quality of life	2.3 ± 0.7	2.2 ± 0.6	2.4 ± 0.7	2.5 ± 0.8	2.3 ± 0.8	2.4 ± 0.7	2.4 ± 0.7	2.5 ± 0.7
SF-36 [mean ± SD]	Physical functioning	65.2 ± 23.6	57.1 ± 28.5	59.3 ± 28.7	58.4 ± 28.9	71.0 ± 22.1	65.7 ± 17.4	64.7 ± 24.6	54.4 ± 25.2*
	Role physical	69.3 ± 26.2	72.3 ± 30.9	60.3 ± 26.0	59.6 ± 26.8	73.0 ± 23.6	62.3 ± 23.3*	65.8 ± 25.9	55.2 ± 28.2*
	Bodily pain	67.0 ± 22.9	63.1 ± 29.7	55.2 ± 26.4	55.2 ± 27.9	65.9 ± 24.1	59.3 ± 28.3	60.6 ± 24.2	48.0 ± 21.0**
	General health perceptions	55.2 ± 20.7	48.0 ± 24.9	49.7 ± 22.6	51.3 ± 22.5	56.9 ± 22.5	50.8 ± 19.6	50.3 ± 22.8	41.2 ± 22.8*
	Vitality	63.9 ± 17.4	64.4 ± 16.6	58.8 ± 19.3	56.6 ± 19.9	65.5 ± 19.2	62.7 ± 19.9	60.1 ± 18.6	55.3 ± 23.1
	Social functioning	77.4 ± 21.0	81.1 ± 23.8	71.2 ± 24.1	71.7 ± 24.2	77.5 ± 22.8	72.8 ± 22.9	76.5 ± 21.0	67.7 ± 25.9*
	Role emotional	72.2 ± 23.9	74.7 ± 30.9	63.7 ± 27.0	62.1 ± 29.6	74.7 ± 22.1	66.4 ± 24.7	66.4 ± 26.0	58.0 ± 28.0
	Mental health	72.7 ± 18.5	77.4 ± 18.9	66.5 ± 19.3	67.7 ± 21.7	71.8 ± 18.1	71.2 ± 18.7	67.1 ± 19.4	62.7 ± 22.3

The COOP/WONCA items were scored on a five-point ordinal scale ranging from 1 to 5 (lower is better), and responses to the SF-36 were calculated the eight subscales from 0 to 100 points (higher is better)

Data are presented as mean ± SD

*P < 0.05; **P < 0.001 between subjects with normal lung functions and those with ventilatory impairment

There are several limitations of this study. Firstly, it is difficult to prove the causal relationship between air pollution and the decrease of HR-QoL in the current study. Although this study includes a health survey in each season and a correlation between air pollution and HR-QoL was observed to some extent, the survey is of a cross-sectional design and the participants of the survey were not continuously followed over a long period. A follow-up study is thus needed to conclusively ascertain the effect of air pollution on HR-QoL. Secondly, the male-to-female ratio in this study was 1:1.75, whereas the male-to-female ratio of Mongolian population between 40 and 79 years old from 2012 to 2013 was 1:0.9 [47, 48]. Non-uniformity in sex balance might be explained by the fact that this study was carried out during the day on weekdays, and that only passers-by participated in the study. Hence, there potentially is selection bias in the sampling for this study although our objective was not to investigate the general population but rather, assess the difference between the specific groups classified by household fuel type, and subjects with and without ventilatory impairment. Finally, we carried out the PMF analysis using only 37 samples (19 in the warm season and 18 in the cold season) of filters. The small number of samples used in the receptor model might lack validity and weaken the reliability of the model solution as the concentration of each species showed daily and seasonal fluctuations. Solid fuel factor, however, was always prominent in the PMF solution even when the factor number was changed from three to seven while the other factors except for soil was uncertain.

## Conclusions

The current study showed that ambient air pollution in Ulaanbaatar worsened in the cold season. The receptor model showed that solid fuel combustion contributed a large part to air pollution in the cold season. Many aspects of HR-QoL in subjects with ventilatory impairment using smoke-rich fuels who were above 40 years of age deteriorated only in the cold season when compared to those with normal lung function. These results suggest that countermeasures or interventions by policymakers to reduce solid fuel usage in winter could improve HR-QoL of the residents of Ulaanbaatar, especially for subjects with ventilatory impairment.

## Additional file

Additional file 1: Table S1. Factors associated with HR-QoL of Mongolian subjects. (DOCX 44 KB)

## Abbreviations

Al: Aluminum
APS: Aerodynamic particle sizer
As: Arsenic
Ba: Barium
BMI: Body mass index
Ca: Calcium
Ca^2+^: Calcium ion
Ce: Cerium
Cl^−^: Chloride
Co: Cobalt
Cr: Chromium
Cs: Cesium
Cu: Copper
EC: Elemental carbon
EPA: Environmental Protection Agency
Fe: Iron
FEV_1_: Forced expiratory volume in one second
FVC: Forced ventilatory capacity
Hf: Hafnium
HR-QoL: Health-related quality of life
K: potassium
K^+^: Potassium ion
La: Lanthanum
Mg^2+^: Magnesium ion
Mn: Manganese
Mo: Molybdenum
Na: Sodium
Na^+^: Sodium ion
NH_4_^+^: Ammonium ion
Ni: Nickel
NO_3_^−^: Nitrate ion
OC: Organic carbon
PAHs: Polyaromatic Hydrocarbons
Pb: Lead
PMF: Positive matrix factorization
PTFE: Polytetrafluoroethylene
Q: Sum of the squared, scaled residuals
Rb: Rubidium
S/N: Signal to noise ratio
Sb: Antimony
Sc: Scandium
Se: Selenium
SF-36,: Short-form 36 health survey
Si: Silicon
Sm: Samarium
SO_4_^2−^: Sulfate ion
Ta: Tantalum
Th: Thorium
Ti: Titanium
V: Vanadium
VC: Vital capacity
W: Tungsten
Zn: Zinc
