# Supplementary material for: Effects of air pollution and seasons on health-related quality of life of Mongolian adults living in Ulaanbaatar: cross-sectional studies
Source: BMC Public Health. 2017 Jun 23;17:594. doi: 10.1186/s12889-017-4507-1 (PMC5481926; doi:10.1186/s12889-017-4507-1)
Supplement: Additional file 1: Table S1. — Factors associated with HR-QoL of Mongolian subjects. (DOCX 44 KB) [file 12889_2017_4507_MOESM1_ESM.docx]

Supplementary table Factors associated with HR-QoL of Mongolian subjects

| COOP/WONCA | Dependent variables | | | | | | | | | | | | | | | |
| --- | --- | --- | --- | --- | --- | --- | --- | --- | --- | --- | --- | --- | --- | --- | --- | --- |
|  | Physical fitness | | Feelings | | Daily activities | | Social activities | | Change of health | | Overall health | | Pain | | Quality of life | |
| Independent variables | Crude  OR | Adjusted  OR | Crude  OR | Adjusted  OR | Crude  OR | Adjusted  OR | Crude  OR | Adjusted  OR | Crude  OR | Adjusted  OR | Crude  OR | Adjusted  OR | Crude  OR | Adjusted  OR | Crude  OR | Adjusted  OR |
| Older age (10 years) | 1.42  (1.22 - 1.66) | 1.41  (1.17 - 1.71) | 0.73  (0.62 - 0.87) | 0.74  (0.60 - 0.90) | 0.80  (0.67 - 0.96) | 0.79  (0.64 - 0.98) | 0.84  (0.72 - 0.99) | 0.89  (0.73 - 1.07) | 1.01  (0.82 - 1.24) | 1.09  (0.85 - 1.41) | 1.00  (0.86 - 1.16) | 1.04  (0.86 - 1.26) | 0.99  (0.84 - 1.16) | 0.98  (0.80 - 1.21) | 0.69  (0.59 - 0.81) | 0.76  (0.63 - 0.92) |
| Male | 0.64  (0.46 - 0.88) | 0.58  (0.38 - 0.89) | 0.63  (0.45 - 0.88) | 0.64  (0.40 - 1.00) | 0.79  (0.55 - 1.13) | 0.71  (0.44 - 1.14) | 0.99  (0.71 - 1.37) | 1.05  (0.69 - 1.61) | 0.77  (0.49 - 1.01) | 0.84  (0.47 - 1.52) | 0.66  (0.48 - 0.91) | 0.58  (0.38 - 0.88) | 0.40  (0.28 - 0.58) | 0.38  (0.24 - 0.61) | 1.07  (0.78 - 1.48) | 0.88  (0.57 - 1.34) |
| Lower BMI (< 25) | 0.86  (0.62 - 1.19) | 1.00  (0.69 - 1.46) | 1.18  (0.85 - 1.65) | 1.24  (0.83 - 1.84) | 1.11  (0.78 - 1.58) | 1.21  (0.79 - 1.84) | 1.43  (1.03 - 1.99) | 1.60  (1.09 - 2.34) | 0.51  (0.31 - 0.83) | 0.44  (0.24 - 0.78) | 0.89  (0.64 - 1.22) | 1.05  (0.72 - 1.53) | 0.92  (0.65 - 1.30) | 1.31  (0.87 - 1.97) | 1.16  (0.84 - 1.61) | 1.17  (0.80 - 1.71) |
| Ventilatory impairment | 1.65  (1.16 - 2.35) | 1.39  (0.93 - 2.08) | 0.85  (0.58 - 1.23) | 0.84  (0.54 - 1.31) | 1.25  (0.85 - 1.83) | 1.23  (0.79 - 1.92) | 0.86  (0.60 - 1.24) | 0.80  (0.52 - 1.21) | 1.14  (0.71 - 1.83) | 1.11  (0.65 - 1.89) | 1.18  (0.83 - 1.67) | 1.02  (0.68 - 1.54) | 1.18  (0.81 - 1.71) | 1.18  (0.76 - 1.81) | 0.97  (0.68 - 1.39) | 1.05  (0.70 - 1.59) |
| Current smoking | 0.73  (0.51 - 1.03) | 0.89  (0.57 - 1.40) | 0.84  (0.58 - 1.21) | 0.84  (0.52 - 1.36) | 1.16  (0.79 - 1.69) | 1.21  (0.74 - 2.00) | 0.82  (0.57 - 1.17) | 0.61  (0.38 - 0.97) | 1.29  (0.82 - 2.04) | 1.59  (0.87 - 2.91) | 0.88  (0.63 - 1.25) | 1.04  (0.66 - 1.62) | 0.55  (0.37 - 0.82) | 0.76  (0.46 - 1.26) | 1.47  (1.03 - 2.08) | 1.32  (0.85 - 2.06) |
| Higher PM2.5 (10 µg/m^3^) | 1.06  (1.01 - 1.12) | 1.12  (1.05 - 1.18) | 1.05  (1.00 - 1.10) | 1.02  (0.96 - 1.09) | 1.01  (0.95 - 1.06) | 0.97  (0.90 - 1.03) | 1.03  (0.97 - 1.08) | 0.99  (0.93 - 1.05) | 0.96  (0.90 - 1.03) | 0.92  (0.85 - 1.00) | 1.01  (0.96 - 1.06) | 1.00  (0.94 - 1.06) | 1.00  (0.94 - 1.05) | 0.98  (0.92 - 1.05) | 1.06  (1.01 - 1.12) | 1.02  (0.96 - 1.08) |
| Smoke-rich household fuel | 1.01  (0.73 - 1.38) | 1.13  (0.80 - 1.61) | 1.20  (0.86 - 1.67) | 1.36  (0.93 - 1.99) | 1.29  (0.90 - 1.84) | 1.33  (0.89 - 1.99) | 1.28  0.92 - 1.77) | 1.33  (0.93 - 1.91) | 1.47  (0.94 - 2.30) | 1.52  (0.92 - 2.49) | 1.28  (0.94 - 1.76) | 1.46  (1.02 - 2.07) | 1.38  (0.98 - 1.95) | 1.58  (1.07 - 2.33) | 1.58  (1.14 - 2.19) | 1.68  (1.17 - 2.41) |
| Self-reported asthma | 1.43  (0.99 - 2.06) | 1.31  (0.83 - 2.09) | 2.29  (1.57 - 3.33) | 2.15  (1.35 - 3.44) | 2.01  (1.36 - 2.96) | 1.48  (0.91 - 2.39) | 1.57  (1.08 - 2.28) | 1.22  (0.77 - 1.92) | 2.33  (1.48 - 3.65) | 1.53  (0.87 - 2.70) | 2.82  (1.91 - 4.18) | 2.15  (1.34 - 3.44) | 2.17  (1.49 - 3.17) | 1.23  (0.76 - 1.97) | 1.37  (0.95 - 1.99) | 1.11  (0.71 - 1.76) |
| Longer time spent outdoors (h) | 0.95  (0.92 - 0.98) | 0.97  (0.94 - 1.01) | 1.01  (0.98 - 1.04) | 0.99  (0.95 - 1.03) | 0.96  (0.93 - 1.00) | 0.95  (0.91 - 0.99) | 1.00  (0.97 - 1.03) | 0.99  (0.95 - 1.02) | 0.97  (0.93 - 1.02) | 0.98  (0.94 - 1.03) | 0.98  (0.95 - 1.02) | 1.00  (0.96 - 1.03) | 0.96  (0.93 - 1.00) | 0.98  (0.94 - 1.02) | 1.02  (0.99 - 1.06) | 1.01  (0.97 - 1.05) |
| Having respiratory symptoms |  |  |  |  |  |  |  |  |  |  |  |  |  |  |  |  |
| Weather affects cough | 0.97  (0.71 - 1.31) | 0.89  (0.61 - 1.29) | 1.76  (1.27 - 2.45) | 1.22  (0.83 - 1.81) | 2.16  (1.51 - 3.09) | 1.83  (1.20 - 2.78) | 2.05  (1.48 - 2.84) | 1.48  (1.01 - 2.15) | 2.17  (1.38 - 3.40) | 1.54  (0.91 - 2.59) | 1.90  (1.39 - 2.59) | 1.37  (0.95 - 1.98) | 2.08  (1.48 - 2.92) | 1.65  (1.11 - 2.47) | 1.67  (1.22 - 2.29) | 1.17  (0.81 - 1.70) |
| Coughing up phlegm without  having a cold | 1.06  (0.78 - 1.45) | 1.15  (0.75 - 1.74) | 1.95  (1.41 - 2.70) | 2.05  (1.32 - 3.17) | 2.07  (1.47 - 2.92) | 1.82  (1.16 - 2.86) | 1.67  (1.22 - 2.30) | 1.38  (0.91 - 2.10) | 1.82  (1.19 - 2.76) | 1.61  (0.92 - 2.79) | 1.68  (1.23 - 2.29) | 1.63  (1.08 - 2.48) | 1.37  (0.98 - 1.90) | 1.31  (0.84 - 2.05) | 1.66  (1.21 - 2.28) | 1.36  (0.90 - 2.07) |
| Coughing up phlegm first thing  in the morning | 1.22  (0.87 - 1.72) | 1.19  (0.74 - 1.91) | 1.38  (0.97 - 1.96) | 0.79  (0.48 - 1.30) | 1.89  (1.31 - 2.72) | 0.99  (0.60 - 1.64) | 1.67  (1.22 - 2.30) | 1.28  (0.80 - 2.05) | 1.81  (1.17 - 2.81) | 1.07  (0.59 - 1.95) | 1.64  (1.16 - 2.32) | 1.08  (0.67 - 1.74) | 1.54  (1.08 - 2.19) | 1.19  (0.72 - 1.97) | 1.44  (1.02 - 2.03) | 1.04  (0.65 - 1.67) |
| Frequent wheeze | 1.01  (0.74 - 1.38) | 0.96  (0.65 - 1.42) | 1.41  (1.02 - 1.95) | 0.88  (0.58 - 1.33) | 1.59  (1.13 - 2.25) | 0.97  (0.63 - 1.48) | 1.66  (1.17 - 2.35) | 1.04  (0.70 - 1.55) | 1.61  (1.06 - 2.46) | 0.86  (0.51 - 1.46) | 1.49  (1.09 - 2.04) | 0.95  (0.64 - 1.40) | 1.49  (1.07 - 2.08) | 1.15  (0.76 - 1.75) | 1.55  (1.13 - 2.13) | 1.12  (0.76 - 1.66) |
| Allergy | 1.00  (0.73 - 1.37) | 1.02  (0.70 - 1.48) | 1.73  (1.25 - 2.40) | 1.25  (0.85 - 1.84) | 1.50  (1.07 - 2.12) | 1.23  (0.82 - 1.86) | 1.40  (1.02 - 1.93) | 1.24  (0.86 - 1.81) | 1.37  (0.90 - 2.09) | 1.21  (0.73 - 1.99) | 1.45  (1.06 - 1.98) | 1.09  (0.76 - 1.58) | 1.34  (0.97 - 1.87) | 1.13  (0.76 - 1.68) | 1.19  (0.86 - 1.63) | 1.04  (0.71 - 1.51) |
| Infectious disease | 0.82  (0.56 - 1.20) | 0.76  (0.49 - 1.18) | 1.87  (1.27 - 2.76) | 1.56  (1.01 - 2.41) | 2.01  (1.35 - 2.98) | 1.57  (1.00 - 2.46) | 1.61  (1.10 - 2.37) | 1.23  (0.80 - 1.88) | 2.37  (1.49 - 3.76) | 2.27  (1.34 - 3.83) | 1.74  (1.18 - 2.56) | 1.47  (0.95 - 2.27) | 1.93  (1.31 - 2.85) | 1.62  (1.04 - 2.52) | 1.95  (1.33 - 2.86) | 1.66  (1.09 - 2.54) |
| SF-36 | Dependent variables | | | | | | | | | | | | | | | |
|  | Physical functioning | | Role physical | | Bodily pain | | General health perceptions | | Vitality | | Social functioning | | Role emotional | | Mental health | |
| Independent variables | Crude  OR | Adjusted  OR | Crude  OR | Adjusted  OR | Crude  OR | Adjusted  OR | Crude  OR | Adjusted  OR | Crude  OR | Adjusted  OR | Crude  OR | Adjusted  OR | Crude  OR | Adjusted  OR | Crude  OR | Adjusted  OR |
| Older age (10 years) | 1.45  (1.23 - 1.69) | 1.42  (1.16 - 1.72) | 1.04  (0.90 - 1.21) | 0.94  (0.78 - 1.14) | 1.12  (0.96 - 1.31) | 1.10  (0.91 - 1.34) | 0.99  (0.85 - 1.15) | 0.98  (0.81 - 1.20) | 0.89  (0.75 - 1.03) | 0.85  (0.70 - 1.03) | 0.88  (0.75 - 1.02) | 0.80  (0.66 - 0.97) | 1.09  (0.93 - 1.27) | 1.09  (0.90 - 1.33) | 0.66  (0.56 - 0.77) | 0.64  (0.53 - 0.78 |
| Male | 0.62  (0.45 - 0.85) | 0.66  (0.42 - 1.01) | 0.95  (0.69 - 1.30) | 0.94  (0.62 - 1.42) | 0.51  (0.37 - 0.71) | 0.35  (0.23 - 0.55) | 0.60  (0.44 - 0.82) | 0.43  (0.28 - 0.67) | 0.76  (0.56 - 1.05) | 0.64  (0.42 - 0.97) | 0.66  (0.48 - 0.92) | 0.60  (0.39 - 0.93) | 0.85  (0.62 - 1.17) | 0.70  (0.46 - 1.07) | 0.68  (0.49 - 0.93) | 0.58  (0.38 - 0.89) |
| Lower BMI (< 25) | 0.63  (0.45 - 0.87) | 0.71  (0.48 - 1.05) | 0.89  (0.64 - 1.22) | 0.98  (0.68 - 1.42) | 1.00  (0.72 - 1.38) | 1.21  (0.82 - 1.78) | 1.03  (0.75 - 1.42) | 1.22  (0.83 - 1.79) | 1.19  (0.86 - 1.65) | 1.26  (0.87 - 1.83) | 1.69  (1.22 - 2.34) | 1.99  (1.36 - 2.91) | 1.00  (0.72 - 1.38) | 1.06  (0.73 - 1.55) | 1.41  (1.02 - 1.95) | 1.64  (1.12 - 2.40) |
| Ventilatory impairment | 1.72  (1.21 - 2.45) | 1.23  (0.81 - 1.85) | 1.44  (1.01 - 2.06) | 1.38  (0.92 - 2.06) | 1.49  (1.04 - 2.12) | 1.34  (0.89 - 2.03) | 1.42  (1.00 - 2.03) | 1.20  (0.79 - 1.83) | 1.11  (0.78 - 1.58) | 0.99  (0.66 - 1.48) | 1.13  (0.79 - 1.61) | 1.08  (0.72 - 1.64) | 1.24  (0.87 - 1.77) | 1.06  (0.71 - 1.60) | 0.74  (0.52 - 1.06) | 0.83  (0.55 - 1.26) |
| Current smoking | 0.76  (0.53 - 1.07) | 0.95  (0.60 - 1.52) | 0.89  (0.63 - 1.26) | 0.82  (0.53 - 1.28) | 0.95  (0.67 - 1.35) | 1.57  (0.99 - 2.51) | 1.12  (0.79 - 1.58) | 1.35  (0.85 - 2.14) | 1.22  (0.87 - 1.73) | 1.35  (0.86 - 2.11) | 0.78  (0.55 - 1.12) | 0.78  (0.50 - 1.24) | 1.08  (0.76 - 1.53) | 1.25  (0.80 - 1.96) | 0.98  (0.69 - 1.39) | 1.01  (0.64 - 1.58) |
| Higher PM2.5 (10 µg/m^3^) | 0.99  (0.94 - 1.04) | 0.98  (0.92 - 1.04) | 0.98  (0.93 - 1.03) | 0.95  (0.90 - 1.01) | 0.96  (0.91 - 1.01) | 0.92  (0.87 - 0.98) | 1.00  (0.95 - 1.05) | 0.98  (0.92 - 1.04) | 1.00  (0.95 - 1.05) | 0.98  (0.92 - 1.04) | 0.99  (0.94 - 1.04) | 0.96  (0.90 - 1.02) | 1.00  (0.95 - 1.05) | 0.97  (0.92 - 1.03) | 1.04  (0.98 - 1.09) | 1.00  (0.94 - 1.06) |
| Smoke-rich household fuel | 1.29  (0.94 - 1.78) | 1.39  (0.97 - 2.00) | 1.68  (1.22 - 2.32) | 1.63  (1.15 - 2.31) | 1.68  (1.22 - 2.31) | 1.97  (1.37 - 2.84) | 1.48  (1.08 - 2.03) | 1.78  (1.24 - 2.55) | 1.55  (1.12 - 2.13) | 1.52  (1.07 - 2.16) | 1.32  (0.95 - 1.83) | 1.30  (0.90 - 1.86) | 1.82  (1.31 - 2.53) | 1.93  (1.35 - 2.76) | 1.76  (1.28 - 2.42) | 1.69  (1.19 - 2.41) |
| Self-reported asthma | 2.14  (1.47 - 3.11) | 1.34  (0.84 - 2.14) | 1.64  (1.13 - 2.39) | 1.27  (0.80 - 2.01) | 1.80  (1.24 - 2.61) | 1.03  (0.65 - 1.65) | 2.28  (1.55 - 3.35) | 1.53  (0.94 - 2.47) | 1.93  (1.33 - 2.80) | 1.54  (0.97 - 2.43) | 1.72  (1.19 - 2.50) | 1.59  (1.00 - 2.51) | 1.86  (1.28 - 2.69) | 1.30  (0.82 - 2.05) | 1.36  (0.93 - 1.97) | 1.25  (0.78 - 2.01) |
| Longer time spent outdoors (h) | 0.93  (0.90 - 0.96) | 0.96  (0.92 - 0.99) | 0.97  (0.94 - 1.00) | 0.97  (0.94 - 1.01) | 0.96  (0.93 - 0.99) | 0.98  (0.95 - 1.02) | 0.96  (0.93 - 0.99) | 0.96  (0.93 - 1.00) | 0.96  (0.93 - 0.99) | 0.96  (0.93 - 1.00) | 0.98  (0.95 - 1.02) | 0.98  (0.95 - 1.02) | 0.97  (0.94 - 1.00) | 0.99  (0.95 - 1.02) | 0.99  (0.96 - 1.02) | 0.97  (0.93 - 1.01) |
| Having respiratory symptoms |  |  |  |  |  |  |  |  |  |  |  |  |  |  |  |  |
| Weather affects cough | 1.70  (1.25 - 2.33) | 1.48  (1.01 - 2.16) | 1.49  (1.09 - 2.03) | 1.25  (0.87 - 1.80) | 1.65  (1.21 - 2.25) | 1.38  (0.95 - 2.02) | 1.53  (1.12 - 2.08) | 0.96  (0.66 - 1.41) | 1.40  (1.03 - 1.90) | 1.03  (0.71 - 1.49) | 1.40  (1.02 - 1.92) | 1.10  (0.76 - 1.61) | 1.63  (1.19 - 2.23) | 1.40  (0.96 - 2.02) | 1.37  (1.01 - 1.87) | 1.11  (0.77 - 1.61) |
| Coughing up phlegm without  having a cold | 1.80  (1.32 - 2.47) | 1.59  (1.04 - 2.43) | 1.71  (1.25 - 2.34) | 1.43  (0.95 - 2.16) | 1.60  (1.17 - 2.18) | 1.61  (1.05 - 2.45) | 1.84  (1.35 - 2.53) | 1.63  (1.07 - 2.50) | 1.70  (1.24 - 2.32) | 1.50  (0.99 - 2.26) | 1.61  (1.18 - 2.22) | 1.69  (1.11 - 2.57) | 1.76  (1.29 - 2.41) | 1.77  (1.17 - 2.68) | 1.32  (0.96 - 1.80) | 1.35  (0.88 - 2.06) |
| Coughing up phlegm first thing  in the morning | 1.94  (1.37 - 2.74) | 1.26  (0.78 - 2.04) | 1.78  (1.26 - 2.52) | 1.19  (0.74 - 1.90) | 1.82  (1.28 - 2.57) | 1.25  (0.77 - 2.03) | 1.92  (1.35 - 2.73) | 1.33  (0.82 - 2.18) | 1.54  (1.10 - 2.18) | 1.02  (0.64 - 1.63) | 1.48  (1.04 - 2.09) | 1.06  (0.66 - 1.71) | 1.77  (1.26 - 2.50) | 1.05  (0.65 - 1.67) | 1.08  (0.77 - 1.53) | 0.87  (0.53 - 1.40) |
| Frequent wheeze | 1.79  (1.31 - 2.46) | 1.40  (0.94 - 2.08) | 1.61  (1.17 - 2.20) | 1.29  (0.88 - 1.89) | 1.51  (1.11 - 2.07) | 1.21  (0.81 - 1.80) | 2.03  (1.48 - 2.79) | 1.55  (1.04 - 2.31) | 1.60  (1.17 - 2.19) | 1.23  (0.83 - 1.80) | 1.20  (0.87 - 1.65) | 0.98  (0.66 - 1.46) | 1.58  (1.15 - 2.17) | 1.17  (0.79 - 1.72) | 1.26  (0.92 - 1.73) | 1.09  (0.73 - 1.62) |
| Allergy | 1.34  (0.98 - 1.84) | 1.07  (0.73 - 1.57) | 1.08  (0.79 - 1.48) | 0.84  (0.58 - 1.21) | 1.41  (1.03 - 1.92) | 1.27  (0.87 - 1.85) | 1.52  (1.11 - 2.08) | 1.13  (0.77 - 1.65) | 1.21  (0.88 - 1.65) | 1.07  (0.74 - 1.54) | 1.14  (0.83 - 1.57) | 0.94  (0.65 - 1.38) | 1.11  (0.81 - 1.52) | 0.91  (0.63 - 1.32) | 1.28  (0.93 - 1.75) | 1.14  (0.78 - 1.66) |
| Infectious disease | 1.48  (1.01 - 2.17) | 1.35  (0.86 - 2.10) | 1.38  (0.94 - 2.03) | 1.18  (0.77 - 1.81) | 1.97  (1.33 - 2.90) | 1.84  (1.18 - 2.87) | 2.16  (1.45 - 3.22) | 1.80  (1.15 - 2.83) | 1.79  (1.22 - 2.63) | 1.61  (1.05 - 2.48) | 1.31  (0.89 - 1.93) | 1.13  (0.73 - 1.73) | 1.67  (1.14 - 2.44) | 1.43  (0.93 - 2.19) | 1.48  (1.00 - 2.18) | 1.27  (0.82 - 1.97) |

The COOP/WONCA items were scored on a five-point ordinal scale ranging from 1 to 5 (lower is better), and responses to the SF-36 were calculated the eight subscales from 0 to 100 points (higher is better). The median value of the scores were used for cutoff point. The COOP/WONCA chart and SF-36 subscale scores were dichotomized as dependent variables (0 for ≤ 50 percentiles of the scores (less affected); 1 for > 50 percentiles (more affected)).
